# International clinical trials setting for rare cancers: organisational and regulatory constraints—the EORTC perspective

**DOI:** 10.3332/ecancer.2013.321

**Published:** 2013-05-21

**Authors:** Emad Shash, Anastassia Negrouk, Sandrine Marreaud, Vassilis Golfinopoulos, Denis Lacombe, Francoise Meunier

**Affiliations:** EORTC, Avenue E. Mounier 83, B-1200 Brussels, Belgium

**Keywords:** rare cancer, clinical trials, international collaboration

## Abstract

Rare diseases are a serious public health problem that presents unique challenges to many countries. There is no internationally accepted definition for rare diseases. Patients suffering from rare cancers often face challenges, including late or incorrect diagnoses, difficulties finding clinical expertise and accessing appropriate treatments, and uncertainty in clinical decision making, with difficult and rare access for these patients to clinical trials. Treatment choice is difficult as little information is available in the literature. In such situations, clinicians will base treatment decisions on retrospective data or case report series with a lower scientific level of evidence than that obtained from randomised controlled clinical trials. The only way forward is clinical trials organisation, but to perform it within rare indications we are always faced with many methodological, regulatory, and organisational challenges, besides stakeholders’ different views, which are not usually concurrent. The aims of the European Organisation for Research and Treatment of Cancer (EORTC) are to develop, conduct, coordinate, and stimulate translational and clinical research in Europe to improve the management of cancer and related problems by increasing survival but also patient quality of life. In particular, extensive and comprehensive research in the field of rare cancers is beyond the means of individual European hospitals and can be best accomplished through the multidisciplinary multinational efforts of basic scientists and clinicians. In this paper, we will present an overview of the clinical research scene for rare cancers and will try to propose possible steps to improve the current situation.

## Introduction

Rare diseases are a serious public health problem that presents unique challenges to many countries. There is no internationally accepted definition. The European Union (EU) defines them as life-threatening or chronically debilitating diseases of low prevalence (less than 5 per 10,000) that require special combined efforts to address them in order to prevent significant morbidity or mortality. This definition first appeared in EU legislation in Regulation (EC) No. 141/2000 of 16 December 1999 on orphan medicinal products [1]. The United States Orphan Drug Act of 1983 defines rare diseases as any disease or condition that affects less than 200,000 people in the country. The Japanese Medicines Act of 1993 definition of rare diseases is conditions affecting no more than 50,000 people in the country [2].

The EU-supported RARECARE project analysed population-based cancer registry data on European patients diagnosed from 1988 to 2002. Based on the RARECARE definition (incidence <6/100,000/year), the estimated annual incidence rate of all rare cancers in Europe was about 108 per 100,000, corresponding to 541,000 new diagnoses annually or 22% of all cancer diagnoses. Five-year relative survival was on average worse for rare cancers (47%) than common cancers (65%). About 4,300,000 patients diagnosed with a rare cancer are living today in the EU, accounting for 24% of the total cancer prevalence [3].

The prevalence of rare diseases may vary among diverse patient populations. An entity that is rare in one patient population may be common in another. For example, several malignancies are considered rare in the paediatric but not in the adult population, because in general, fewer children develop cancer compared with adults.

Patients suffering from rare cancers often face challenges including late or incorrect diagnoses; difficulties finding clinical expertise and accessing appropriate treatments; uncertainty in clinical decision making with rare access to clinical trials. Treatment choice is difficult when the patient is diagnosed with a rare disease or a pathological or molecular pattern, and little information is available in the literature. In such situations, clinicians will base treatment decisions on retrospective data or case report series with a lower scientific level of evidence than that obtained from randomised controlled clinical trials. We present an overview of the clinical research scene for rare cancers and propose possible steps to improve the current situation.

## Challenges

### Methodological challenges

The main challenge in performing appropriate clinical trials for rare cancers is the small number of patients affected and therefore available for clinical research. Observational retrospective series or single-arm clinical trials often represent the highest level of evidence available. Most single centres or national clinical trial consortiums cannot accrue the number of patients required for a robust randomised controlled trial in a reasonable amount of time.

In order to reduce the number of patients required, oncology trials are often designed using surrogate endpoints, such as tumour response rate or progression-free survival as a pragmatic solution, even though these do not always correlate adequately with clinically meaningful endpoints, namely the duration and quality of patients’ life. The United States Food and Drug Administration (FDA) established the drug approval regulation in a way that the unmet medical needs could be addressed using acquired evidence that is reasonably likely to predict clinical benefit. Sponsors of drugs that are granted accelerated approval are required to conduct postapproval clinical trials to verify clinical benefit and thereby retain the drug’s marketing indication [4]. Similar strategies are applicable for medicinal approval within the EU as regulated by the European Medicines Agency (EMA) [5].

Hodgkin’s lymphoma conforms to the definition of rare disease, and there is no approved standard of care in the refractory setting. On 19 August 2011, the FDA granted accelerated approval to brentuximab vedotin (Adcetris™ for Injection, made by Seattle Genetics, Inc.) [4] for two indications: treatment of patients with Hodgkin lymphoma after failure of autologous stem cell transplant (ASCT) or after failure of at least two prior multi-agent chemotherapy regimens in patients who are not candidates for ASCT, and treatment of patients with systemic anaplastic large cell lymphoma (ALCL) after failure of at least one prior multi-agent chemotherapy regimen. The accelerated approval for Hodgkin lymphoma was based on a single-arm multicentre clinical trial that enrolled 102 patients who had cluster of differentiation 30 (CD30)-positive Hodgkin lymphoma that relapsed after ASCT. The primary efficacy endpoint, the objective response rate of brentuximab vedotin as a single agent as determined by independent review, was 73% (95% CI 65%–83%) with a median duration of 6.7 months (95% CI 4–14.8).The complete remission rate was 32% (95% CI 23.3%–42.3%) with a median duration of 20.5 months (95% CI 12–not reached) [6].

Later, the FDA granted accelerated approval for systemic ALCL based on a single-arm multicentre clinical trial (SG035-0004) that enrolled 58 patients who had CD30-positive systemic ALCL and had previously received front-line multi-agent chemotherapy. The primary efficacy endpoint, objective response rate by independent review, was 86% (95% CI 77%–95%) with a median duration of 12.6 months. Complete remission rate was 57% (95% CI 44%–70%) with a median duration of 13.2 months [7]. The EMA granted accelerated approval for brentuximab vedotin for the treatment of both indications, and on 11 January 2012, the European Commission granted orphan designation (EU/3/11/939) to Takeda Global Research and Development Centre (Europe) Ltd, United Kingdom [5].

In another example, on 6 October 2006, the FDA granted approval to vorinostat (Zolinza™, Merck & Co., Inc.), a histone deacetylase inhibitor, for the treatment of cutaneous manifestations of cutaneous T-cell lymphoma (CTCL) in patients with progressive, persistent, or recurrent disease during, or following two systemic therapies. Data supporting approval came from a single-arm open-label trial conducted at 18 centres in the US and Canada that enrolled 74 patients with stage IB and higher CTCL who had failed two systemic therapies (one of which must have contained bexarotene). On 13 February 2009, Merck Sharp & Dohme Limited (MSD) officially notified the Committee for Medicinal Products for Human Use (CHMP) that it wished to withdraw its marketing authorisation application for Vorinostat for the treatment of adult advanced CTCL after the committee expressed concern over the design of the main study. Specifically, the safety and effectiveness of Vorinostat could not be adequately assessed, because it was not compared with any other treatment. The study did not use an overall survival endpoint and CHMP was concerned about the risk of thromboembolic events (problems caused by the formation of clots in the blood vessels) in patients taking Vorinostat MSD. Therefore, at the time of withdrawal, the Committee’s view was that a benefit of Vorinostat MSD had not been sufficiently demonstrated, and any benefits did not outweigh the identified risks [4, 6].

It is obvious from the examples above that obtaining accelerated approval by single-arm or randomised phase II trials could be the first step towards marketing a drug for a rare cancer. Additional effort is often needed to retain the marketing indication, as eventually a level of evidence comparable with common cancers has to be demonstrated. The advantage of accelerated approval is therefore temporary and can prove damaging to the pharmaceutical company if the cost of development is not recuperated during the period of authorisation.

Appropriate and timely diagnosis and identification of eligible patients is an additional barrier to clinical trials. Indeed, rare cancers are frequently more difficult to diagnose and not all countries have an established network of reference centres or efficient processes for patient referral. Therefore, frequently a diagnosis is late or incorrect. The accurate determination of histological diagnosis is a critical factor for the treatment strategies. Sarcomas, a heterogeneous group of tumours, perfectly illustrate this paradigm. Their incidence is about 2% [8], and as shown by Ray-Coquard *et al *[9], more than 40% of histological diagnoses are modified after review. Clinical practice guidelines for their management have been established; however, non-adherence to these guidelines remains high [10].

### Organisational challenges

The relatively small number of patients available for randomised controlled trials can be addressed by building multi-centre national or international networks of institutions. However, intergroup studies present additional challenges since multiple parties are needed, and these must agree both on a specific project and on how to work together to successfully complete the trials. In such a setting, operational difficulties, although not specific to rare cancer trials, are further amplified due to the scale of the projects.

We can identify specific problems common to most intergroup rare cancer trials. Cost can increase disproportionately to the number of enrolled patients and become a burden for trials that are already underfunded. Practical issues such as ensuring reliable communication among sponsors, laboratories, and investigators; timely drug delivery at clinical sites and adequate monitoring and follow-up can even represent a source of bias for the results of the clinical trial. By definition, most centres will only enroll a small number of patients, so maintaining adequate quality of care becomes nearly impossible.

Cooperation between different networks is further complicated when a multi-disciplinary approach is required. Phase III clinical trials may involve multiple treatment modalities (e.g. surgery or radiotherapy), central pathology or imaging review, integrated translational research, quality of life assessment or quality assurance. In such cases, a lot of preparatory work is necessary among the involved networks in order to have a homogeneous approach to all of these aspects, yet the resources for such work are often unavailable.

### Regulatory challenges

Regulatory procedures, such as ethics committee submission, patient information sheet and informed consent creation and approval, clinical centre activation, insurance acquisition, human and data protection compliance and investigator reimbursement, differ across national borders and place additional burden on an international intergroup trial. Moreover, most of these procedures must be repeated when a protocol is amended. The legal framework is also different, so that the same clinical trial can vary between the various participating countries.

Even after completion of a successful clinical trial, the extension of indication for anti-cancer agents requires additional assumptions. Pharmaceutical companies develop, manufacture and market drugs, and the priorities of industry are defined by the potential market. Therefore, even taking into account advantages put in place by regulators for orphan indications, the return on the investment for many rare indications is not sufficient to justify label extension. On the other hand, academia performs clinical trials that sometimes result in new standards of care for rare cancers. Unfortunately, there is no defined procedure for academia to request or EMA to directly extend drug indications. Consequently, off-label indications are frequently considered standard treatment for rare cancers.

Some steps have been taken to address this problem. For example, French legislation foresees a possibility for competent authorities to deliver a recommendation for temporary use (Recommandations Temporaires d’Utilisation) [11-13] to allow the use of medication outside registered indications for a duration of three years based on scientific evidence for rare diseases. Still, only the marketing authorisation holder can expand the indication permanently. If the relevant application does not take place, many rare cancers are treated with off-label standard treatment.

## Potential solutions and role of Academic Research Networks for such a ‘niche’ population

We can identify approaches to overcome the barriers in clinical research for rare cancers. Well-designed and monitored observational studies may provide the necessary evidence for the approval of off-label drugs or for extension of marketing authorisation. Integration of a quality of life component is necessary to introduce treatments that improve patients’ ability to function or reject treatments that are too toxic for the benefit they provide. Careful choice of clinically relevant endpoints may reduce the probability of inconclusive clinical trials. Novel statistical designs might present a partial solution by reducing the number of patients needed to answer clinical questions. Adaptive designs that allow modifications to the trial and/or statistical procedures of the trial after its initiation without undermining its validity and integrity might enable researchers to reach robust conclusions or disregard unsuccessful efforts and make clinical trials more flexible, efficient and fast [14]. However, a large initial investment is needed to apply adaptive methods, and the expertise needed has to be built gradually and cautiously [15].

Innovative operational solutions might make clinical trials for some indications feasible by reducing the barrier to initiate such trials. An attractive approach would be to group together several research projects with a common denominator, for example a cancer type or expression of a particular molecular biomarker or application of a treatment modality. Patients could be screened and then offered an available clinical trial or a translational research project or be followed-up until further treatment options become available; these options could be available in the patient’s local hospital or in a local or distant reference centre. In this way, all rare cancer patients would potentially have access to clinical trials. Such a platform could answer clinical questions and increase the understanding of the disease in a shorter time and with lower cost. It is of value to note that most of the barriers to research we have identified in this review are resolvable via a platform approach; the reason is that the responses to these challenges are grouped and tackled in advance and in a systematic way for different trials. A model for this innovative platform might be the International Rare Cancer Initiative (IRCI), a joint initiative between the European Organisation for Research and Treatment of Cancer (EORTC), the US National Cancer Institute (NCI), the National Institute for Health Research (NIHR) Cancer Research Network (NCRN) and Cancer Research UK (CR-UK). The objective of this initiative is to facilitate the development of international clinical trials for patients with rare cancers in order to boost the progress of new treatments for these patients. The initiative hopes to encourage the use of innovative methodologies to maximise the potential for answering research questions and to identify and overcome barriers to international trials to allow agreed IRCI trials to run smoothly [16].

Regulatory issues have to be addressed in order for substantial change to happen. Both EMA and FDA are thinking along these lines, but the decisions required are not yet in place and legal requirements change slowly. Conditional approval might be followed by phase IV clinical data, registry data, information from payers, hospitals and consortiums, and not necessarily by formal phase III clinical trial data. Extension of approval for rare disease treatments might be granted without application from the manufacturer, if reliable evidence of efficacy, toxicity and cost is available. Until these issues are addressed, consultation with central regulatory authorities is advised for investigators wishing to embark on a clinical study for rare cancers.

Change cannot take place without extensive dialogue among all the stakeholders involved in the rare cancer research scene. Regulatory authorities and pharmaceutical companies should be part of the solution. The role of academic research organisations to enable a productive relationship between the public and the private healthcare sectors has been widely recognised [17, 18]. Patient advocacy organisations are increasingly active in promoting the interests of patients worldwide, and they can apply pressure to all involved public and private parties for an improved research environment for their disease [19].

## Conclusion and future directions

We presented the difficulties that investigators face in clinical research for rare cancers in so far as methodological, operational and regulatory issues are concerned. Most are the result of the failure to recognise that research for rare diseases cannot be conducted in the same way as more common clinical entities. The same framework that provides scientific rigor and quality control in clinical research for common diseases is obstructing discovery in rare conditions. Wider communication of the problem and coordinated effort from investigators, local ethics committees, regulators and patient organisations are needed. Frequent diseases are being fragmented by molecular sub-entities, and the issues developed herein may no longer remain specific to rare diseases. Therefore, there are reasons to consider revising the entire research environment. Rare diseases offer an opportunity and a possible template to prepare our future healthcare system in the current economical environment.

## Conflict of Interest

Nothing to declare
